# Minimum Entropy Production Effect on a Quantum Scale

**DOI:** 10.3390/e23101350

**Published:** 2021-10-15

**Authors:** Ferenc Márkus, Katalin Gambár

**Affiliations:** 1 Department of Physics, Budapest University of Technology and Economics, Budafoki út 8, H-1111 Budapest, Hungary; 2 Institute of Microelectronics and Technology, Kálmán Kandó Faculty of Electrical Engineering, Óbuda University, Tavaszmező u. 17, H-1084 Budapest, Hungary; gambar.katalin@uni-obuda.hu; 3 Department of Natural Sciences, National University of Public Service, Ludovika tér 2, H-1083 Budapest, Hungary

**Keywords:** Landauer’s principle, quantized electric and thermal conductances, quantized entropy current, least action principle, minimal entropy transfer

## Abstract

The discovery of quantized electric conductance by the group of van Wees in 1988 was a major breakthrough in physics. A decade later, the group of Schwab has proven the existence of quantized thermal conductance. Advancing from these and many other aspects of the quantized conductances in other phenomena of nature, the concept of quantized entropy current can be established and it eases the description of a transferred quantized energy package. This might yield a universal transport behavior of the microscopic world. During the transfer of a single energy quantum, *hν*, between two neighboring domains, the minimum entropy increment is calculated. It is pointed out that the possible existence of the minimal entropy transfer can be formulated. Moreover, as a new result, it is proved that this minimal entropy transfer principle is equivalent to the Lagrangian description of thermodynamics.

## 1. Introduction

Many concurrent research areas are relying on quantized conductances, such as electric [1], thermal [2,3,4], integer [5], fractional [6,7], and spin quantum Hall effect [8]. A large number of papers focus on half-integer quantum Hall effect in topological insulators [9], and in the quantized light–matter interaction on the edge state of a quantum spin Hall insulator [8]. Furthermore, the electron transport through individual molecules as a candidate for future nanoelectronic circuits also exhibits similar interesting properties [10,11,12].

The transport phenomena have a quantum origin on the microscopic level and, it seems, the relevant conductances reflect their quantized behavior. Based on this approach and following the idea of quantized electric and thermal conductances, we introduce the quantized entropy conductance. This step enables us to incorporate the thermodynamic concepts into quantum processes. We show that the entropy change during the transfer of an energy package of hν can be expressed. The description allows us to formulate a mathematical equation that expresses the minimal entropy production principle for the microscopic world. These results may be useful to acquire the maximal physical limits of reversibility of coherent quantum systems, e.g., in quantum dots and quantum computers, relying on these.

The discussed theory incorporates various fields of physics. To aid the reader, we provide a brief summary of the phenomena used. Afterwards, a coherent frame arises in which the main aims and the applicable methods are discussed.

## 2. Historical Considerations

### 2.1. The Quantized Electric Conductance

**Figure 1:**
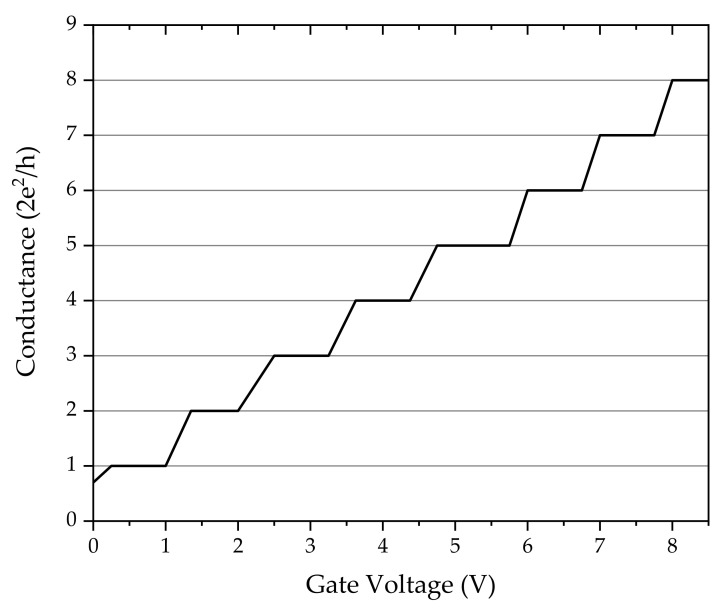
The electric conductance showed as a function of the gate voltage. The channel width, *w*, can be modulated by the gate voltage. The quantized behavior can be read out directly from the figure. The quantum of electric conductance is $2e^{2}/h$ based on theoretical predictions. Inspired by the work of van Wees et al. [1].

First, a short introduction to quantized electric conductance is given below. Landauer [13,14,15] theoretically predicted the existence and the amount of the quantized electric conductance which can be expressed as

(1) $$G=\frac{2e^{2}}{h}=7.75\cdot 10^{-5}\;S,$$

where *e* is the elementary charge and *h* is the Planck constant. The factor of 2 appears due to the two spin states of the electron. In general

(2) $$G=R^{-1}=\sigma A/L,$$

where *R* is the ohmic resistance, σ is the specific electric conductivity, *A* is the cross-section and *L* is the length of the conductor. The experimental proof for the theoretical prediction was elaborated by van Wees et al. [1]. Theoretically, the quantized conductance may appear in a nanowire with a width, *w*, comparable to the length of Fermi wavelength $\lambda_{F}$, and the length, *L*, which is less than the mean free path of electrons. The quantized electric conductance was measured in a 2D electron gas realized within an AlGaAs–GaAs boundary layer. The gate voltage dependence of the electric conductance is shown in Figure 1 (or in Figure 2 of ref. [1]).

The channel width, *w*, can be modulated by the gate voltage. The quantized behavior can be read out directly from the figure. The quantum of electric conductance is $2e^{2}/h$ in agreement with the theoretical predictions.

**Figure 2:**
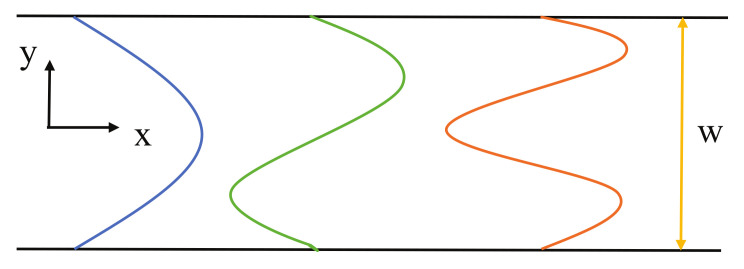
The propagating plane wave in the direction *x* and the cross-modes in the direction *y* in the 2D waveguide.

The confined electrons, present in a long, straight 2D quantum wire can be described by the following wave function:

(3) $$\Psi_{k,j}\left(x,y\right)∼\sin\left(ikx\right)\sin\left(\frac{j\pi }{w}y\right),$$

where *k* is the wavenumber, *j* is an integer quantum number (depicted in Figure 2).

The first factor pertains to the propagating plane wave in the direction *x*, while the second factor describes the cross-modes in the direction *y*. The corresponding energy to the wave function $\Psi_{k,j}\left(x,y\right)$ expresses a continuum spectrum arising from the *x*-axis solution, while it contains quantized levels, due to the finite width, *w*, in the *y*-direction [16]:

(4) $$\varepsilon \left(k,j\right)=\frac{\hbar^{2}k^{2}}{2m}+\frac{\hbar^{2}\pi^{2}}{2mw^{2}}j^{2}.$$

The number of quantized states ε(k,j) below the Fermi surface is $N∼2w/\lambda_{F}$. Moreover, if the thermal energy can be neglected compared to the chemical potential difference $\Delta \mu \gg k_{B}T$ between the contacts, the electric current of the *j*th channel can be expressed as

(5) $$I_{j}=ev_{j}{\left(\frac{dn}{dE}\right)}_{j}\Delta \mu =e^{2}v_{j}{\left(\frac{dn}{dE}\right)}_{j}V,$$

where $v_{j}$ is the propagation velocity along the direction *y*, $dn/{dE}_{j}$ is the density of states at the Fermi level for the *j*th state, and *V* is the voltage difference V=Δμ/e. The number of states between *k* and k+dk in one dimension for unit length is

(6) $$\frac{dn}{dk}=\frac{1}{2\pi },$$

by which the density of states can be obtained as

(7) $${\left(\frac{dn}{dE}\right)}_{j}={\left(\frac{dn}{dk}\frac{dk}{dE}\right)}_{j}=\frac{2}{hv_{j}},$$

where the factor of 2 arises from the spin degeneracy. The total current

(8) $$I=\sum_{j=1}^{N}I_{j}=\frac{2e^{2}}{h}NV$$

can be expressed, where *N* is the number of channels. The quantized electric conductance can be read out from the formula.

### 2.2. The Quantized Thermal Conductance

Based on thermodynamic and information theory assumptions, Pendry—analogously to the Landauer’s formula—intuitively predicted the expression of the maximal rate of cooling (transferable energy, *Q*, per unit time giving the quantum limits for information flow) for one channel [17] as

(9) $$\frac{dQ}{dt}\leq \frac{\pi k_{B}^{2}T^{2}}{3\hbar },$$

where $k_{B}$ is the Boltzmann constant, *ℏ* is the reduced Planck constant, and *T* is the temperature. Dividing by *T*, the maximal entropy current (dS/dt) in one channel can be obtained as

(10) $$\frac{dS}{dt}\leq \frac{\pi k_{B}^{2}T}{3\hbar }.$$

Later, Rego and Kirczenow [18] deduced the thermal conductance of a quantum wire using more sophisticated calculations. Their result is

(11) $$\Lambda =\frac{\pi^{2}k_{B}^{2}T}{3h}.$$

Here, the Λ notation is introduced for the quantum of thermal conductance. Comparing Equations (10) and (11) a factor 2 difference appears. Furthermore, according to Equation (6) the maximal entropy change and the quantized thermal conductance are related to each other. The origin of quantized thermal conductivity is explored by many theoretical groups from various viewpoints [19,20,21,22,23].

Another consideration based on the Drude–Lorentz theory also hints at the existence of quantized thermal conductance. In the model, the relation between the λ thermal conductivity and σ electric conductivity can be expressed as

(12) $$\lambda =\frac{\pi^{2}}{3}{\left(\frac{k_{B}}{e}\right)}^{2}T\sigma ,$$

where σ is the specific electric conductivity [24]. However, in semiconductors the phonons are responsible for heat conduction, thus the expression of $G=e^{2}/h$ holds. Using the form for the electric conductivity described in Equation (2), the following expression for the thermal conductivity can be derived:

(13) $$\lambda =\frac{\pi^{2}k_{B}^{2}T}{3h}\frac{L}{A}.$$

This might look only a formal analogy, since the quantized thermal measurements were elaborated in semiconductors, while the Drude–Lorentz model is formulated to describe conducting electrons. However, as it is experimentally verified, the thermal conductance also displays the quantized behavior, despite the different carriers. Thus, similarly to the quantized electric conductance, the quantized thermal conductance of

(14) $$\Lambda =\lambda \frac{A}{L}=\frac{\pi^{2}k_{B}^{2}T}{3h}=9.46\cdot 10^{-13}\;T\;\left[\frac{W}{K}\right]$$

can be obtained. Here, the temperature dependence of the conductance destroys the feeling that conductance should depend only on universal quantities. The question arises as to whether we could not find a similarly suitable thermodynamic conductance that now depends only on universal quantities. After all, from thermodynamic considerations, this may suggest that the thermal conductance might have a close relationship with the quantized behavior of the entropy current. If this is the case, we would expect the previously mentioned requirement of universality.

**Figure 3:**
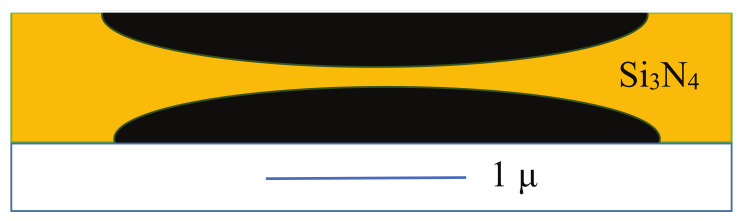
Experimental realization of a Si${}_{3}$N${}_{4}$ thermal waveguide [2]. Physical dimensions: length: L∼1μm; width: w=200 nm; layer thickness: d=60 nm.

The quantized thermal conductance was first measured in a Si${}_{3}$N${}_{4}$ waveguide (Figure 3 or see a photo in Figure 1c of ref. [2]) by Schwab et al.

The obtained result for the thermal conductivity in the temperature range of 60 mK to 6 K is shown in their article. Both the experimental setup and the measurement technique are fascinatingly sophisticated, as depicted in Figure 1a–c in ref. [2].

The quantum of thermal conductance based on theoretical considerations is

(15) $$g_{0}=\frac{\pi^{2}k_{B}^{2}T}{3h}.$$

**Figure 4:**
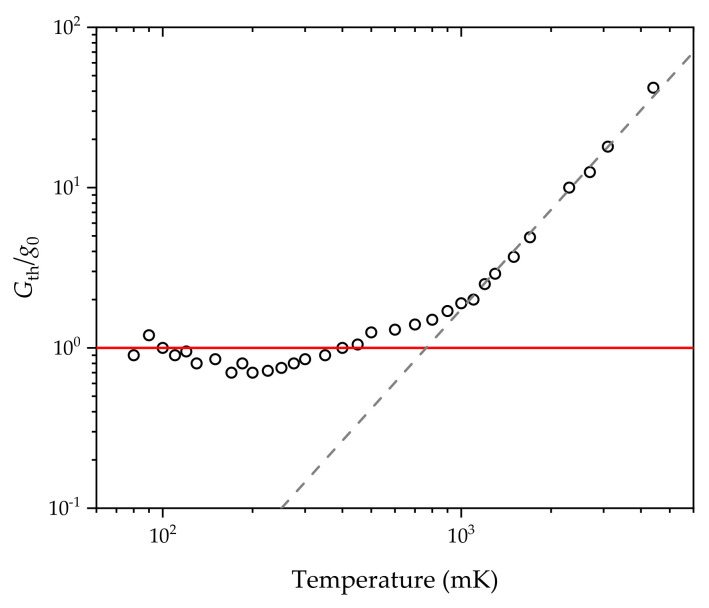
The quantized behavior of thermal conductance [2,3,4]. The appeared plateau can be well recognized in the temperature range 60–700 mK.

Taking into account that in the measurement, four waveguides are present and each is expected to carry just four populated modes below the critical temperature of

(16) $$T<T_{c}=\frac{\pi \hbar v}{k_{B}w}=0.8\;K,$$

the thermal conductance of the arrangement should approach a limiting value of $16g_{0}$. In the experiment, the width of the channel was 200 nm, while v=6000 m/s is the speed of sound in the material. The measurement data, normalized by $16g_{0}$ [2,3,4] is presented in Figure 4 (or in Figure 3 of ref. [2]).

Please note that below 700 mK, data points significantly deviate from the linear fashion and converge to the value of $16g_{0}$. The appeared plateau can be well recognized in the temperature range 60–700 mK. The constant thermal conductivity of $16g_{0}$ in the temperature range of 60–700 mK proves the quantized behavior of the thermal conductance.

### 2.3. Lagrangian Description of Heat Conduction

The quantum limit for information flow raised by Pendry is presumably related to an extreme-value problem. If so, it is probable that it is inherited from its thermodynamic background. To investigate, it is worth reviewing the extremum principle formulated for thermodynamics and the generating potential φ introduced into the principle. The significance of these will be highlighted below.

The theory is based on the least action principle

(17) $$S_{\mathrm{action}}=\int_{t_{1}}^{t_{2}}L\;dt=\mathrm{extremum},$$

where *L* is the Lagrange function of the problem. To proceed, the principle has to be applied for the Fourier equation for heat conduction, which is a constant coefficient linear parabolic differential equation for temperature *T*

(18) $$\varrho c_{v}\frac{\partial T}{\partial t}-\lambda \nabla^{2}T=0,$$

and which equation cannot be derived directly from the Hamiltonian principle for temperature *T*. Here, λ is the thermal conductivity, introduced earlier, $c_{v}$ is the specific heat, and ϱ denotes the mass density. Assume that a potential space, φ, exists, which produces a measurable local equilibrium (classical) temperature field as follows:

(19) $$T\left(x,y,z,t\right)-T_{0}=-\frac{\partial \varphi }{\partial t}-\frac{\lambda }{\varrho c_{v}}\nabla^{2}\varphi =-\frac{\partial \varphi }{\partial t}-D\nabla^{2}\varphi ,$$

where $T_{0}$ is a reference temperature. The presence of this reference temperature grants that the potential φ has at least one well-defined zero value and does not increase beyond all limits, e.g., limited from above. To simplify notation, it is worth introducing thermal diffusivity as

(20) $$D=\frac{\lambda }{\varrho c_{v}}.$$

Substituting the expression from Equation (19) into the equation of heat conduction in Equation (18), the equation of motion of the problem can be obtained by the potential function of φ as

(21) $$0=-\frac{\partial^{2}\varphi }{\partial t^{2}}+D^{2}\nabla^{2}\left(\nabla^{2}\varphi \right).$$

The equation of motion (field equation) described in Equation (21) is the Euler–Lagrange equation of the heat conduction problem. The equation contains only self-adjoint operators and thus it can be deduced from the following Lagrangian [25,26,27]

(22) $$L=\frac{1}{2}{\left(\frac{\partial \varphi }{\partial t}\right)}^{2}+\frac{1}{2}D^{2}{\left(\nabla^{2}\varphi \right)}^{2}.$$

The presented method is also a good example of how Hamilton’s principle can be applied to dissipative processes [28].

The Lagrangian theory can be quantized and the arising energy packets can be assigned to the thermal propagation [29]. Performing the energy transfer calculations by the first energy level for a silicon film with a width of 100 nm and a cross-section of $10^{-6}$ m${}^{2}$ at the temperature of T=80 mK, the obtained energy is $\varepsilon_{1}=7.0\times 10^{-14}$ J = $4.4\times 10^{5}$ eV [30]. This energy value agrees well with the value of the transferred energy per unit time per unit temperature $\varepsilon =7.6\times 10^{-14}$ J = $4.7\times 10^{5}$ eV calculated from the previously mentioned experimental results for silicon nitride in Schwab et al. [2,3,4]. These results prove the material independence of quantized thermal conductance.

## 3. The Quantized Behavior of the Conductance of Entropy Current and the Entropy Production

The change of an extensive physical quantity in a volume depends on the in- or outgoing flow via the total surface of the considered volume and the production or loss of the quantity within the volume. If the extensive quantity is the entropy, *S*, then the balance equation reads as

(23) $$\frac{dS}{dt}=-I_{S}+\Sigma ,$$

where $I_{S}$ is the entropy current and Σ is the entropy production.

Upon thermal propagation, the relation between the entropy current density $J_{s}$ ($J_{s}=I_{s}/A$) and the heat current density can be expressed as usual. Applying Fourier’s law yields

(24) $$J_{q}=-\lambda \nabla T.$$

With the use of Equation (24), the formulation of the entropy current density, $J_{s}$, in the presence of heat transfer can be made:

(25) $$J_{s}=\frac{J_{q}}{T}=-\frac{\lambda \nabla T}{T}=-\frac{\lambda }{T}\nabla T.$$

At this point, we take the form of the quantized thermal conductivity given by Equation (14). Similarly to Λ,

(26) $$\Lambda_{s}=\frac{\Lambda }{T}=\frac{\pi^{2}k_{B}^{2}}{3h}=9.46\cdot 10^{-13}\;\frac{J/K}{K\;s}$$

can be introduced as the quantized entropy conductance. This denotes the entropy flow per unit time and unit temperature. For a given temperature difference of dT, the entropy current is

(27) $$I_{S}=-\Lambda_{s}dT.$$

Recalling the relation from Equation (19)—and neglecting the $D\nabla^{2}T$ term—yields

(28) $$dT=T-T_{0}∼-\frac{\partial \varphi }{\partial t}.$$

In the present form, the expression is integrable, by which the transferred entropy $S_{\mathrm{tr}}$ can be expressed by the potential φ as

(29) $$S_{\mathrm{tr}}=\Lambda_{s}\left(\varphi -\varphi_{0}\right)=\frac{\pi^{2}k_{B}^{2}}{3h}\left(\varphi -\varphi_{0}\right).$$

At this point, it is noted that the potential difference drives the system to equilibrium, and it leads to entropy change. This gives us a deeper meaning that the coefficient $\Lambda_{s}$ is the quantum of entropy conductance.

On the other hand, if dT is related to the transmission of an energy packet, then using the relation of $\varepsilon =k_{B}dT$ yields that the entropy current can be formulated as

(30) $$I_{S}=\frac{\Lambda_{s}}{k_{B}}\varepsilon =\frac{\pi^{2}k_{B}}{3h}\varepsilon .$$

If the energy package is a single quantum with the energy of ε=hν (e.g., a phonon or a photon) then the entropy current carried by it is

(31) $$I_{S}=\frac{\pi^{2}k_{B}}{3}\nu ,$$

where ν is the characteristic frequency of the particle or elementary excitation.

To proceed, it is required to formulate the entropy production density upon thermal transfer [31,32,33]:

(32) $$\sigma_{\mathrm{ep}}=J_{q}\nabla \frac{1}{T}=\lambda {\left(\frac{\nabla T}{T}\right)}^{2}.$$

This can be readily reformulated in quantized form using Equations (25) and (13). The obtained result is

(33) $$\sigma_{\mathrm{ep}}=\frac{J_{s}^{2}}{\lambda }=\frac{1}{T}\frac{\pi^{2}\varepsilon^{2}}{3h}\frac{1}{AL}.$$

Multiplying this equation by the volume V=AL and introducing the entropy production $\Sigma =\sigma_{\mathrm{ep}}V$ of the considered system, the entropy production is thus

(34) $$\Sigma =\frac{1}{T}\frac{\pi^{2}\varepsilon^{2}}{3h}.$$

If the energy transfer can be expressed by an energy packet (or a quasiparticle) with frequency ν, then for the entropy production the following relation holds:

(35) $$\Sigma =\frac{1}{T}\frac{\pi^{2}}{3}h\nu^{2}.$$

The dependence of entropy production on the square of the frequency is remarkable. Nature seems to prefer the scattering of larger energy packets.

## 4. Examples and Applications

Examples are given below on how the use of quantized thermodynamic conductances can be exploited.

### 4.1. Entropy Change during a Single Quantum Transfer

**Figure 5:**
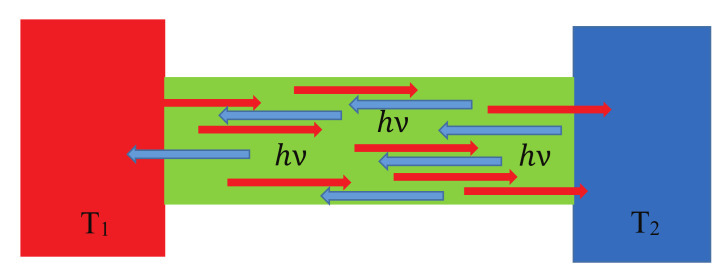
The same energy phonons travel in both directions, but the probability of emission is greater from the hotter domain 1 with $T_{1}$ towards the colder one 2 with $T_{2}$ than vice versa due to the greater population of hν quanta.

Let us consider two subdomains 1 and 2 with equilibrium temperatures $T_{1}$ and $T_{2}<T_{1}$, respectively, as is shown in Figure 5.

Between the two subdomains, a spontaneous hν energy transfer is taken into account. If the energy packet is created in subdomain 1, by which undergoes an entropy production of

(36) $$\Sigma_{1}=-\frac{1}{T_{1}}\frac{\pi^{2}}{3}h\nu^{2}.$$

The negative sign is due to the formation of the quantum. The generated wave packet leaves domain 1, which has an entropy current of

(37) $$I_{S_{1}}=-\frac{\pi^{2}k_{B}}{3}\nu$$

originated from domain 1. Thus, the total entropy decrease in domain 1 holds

(38) $$\frac{dS_{1}}{dt}=-\frac{\pi^{2}k_{B}}{3}\nu -\frac{1}{T_{1}}\frac{\pi^{2}}{3}h\nu^{2}.$$

The energy packet arrives at domain 2, which yields an entropy current income of

(39) $$I_{S_{2}}=\frac{\pi^{2}k_{B}}{3}\nu .$$

On the other hand, the energy packet is spread in the volume causing an entropy production

(40) $$\Sigma_{2}=\frac{1}{T_{2}}\frac{\pi^{2}}{3}h\nu^{2}$$

during this dissipation process. Consequently, the total entropy increase in domain 2 is:

(41) $$\frac{dS_{2}}{dt}=\frac{\pi^{2}k_{B}}{3}\nu +\frac{1}{T_{2}}\frac{\pi^{2}}{3}h\nu^{2}.$$

The total entropy increase in the volume containing 1+2 is thus:

(42) $$\frac{dS}{dt}=\frac{dS_{1}}{dt}+\frac{dS_{2}}{dt}=\left(-\frac{1}{T_{1}}+\frac{1}{T_{2}}\right)\frac{\pi^{2}}{3}h\nu^{2}>0,$$

satisfying the second law of thermodynamics, as expected, because the condition of $T_{2}<T_{1}$ is assumed, e.g., the quantum is emitted from the warmer subsystem. During the formation of the quantum, the entropy decreases in subdomain 1 at temperature $T_{1}$. During the absorption, the entropy increases in subdomain 2 at $T_{2}$. Since $T_{1}>T_{2}$, the net entropy change is positive. If $T_{1}=T_{2}$, e.g., in the thermal equilibrium, no further entropy is produced. The entropy current is independent of the temperature, so the transfer process has no further contribution to the entropy increase. The reversed process, when a quantum is emitted from the cooler subdomain and absorbed in the hotter subdomain, is also statistically possible as enabled by statistical fluctuations. This will yield a negative entropy production for a single quantum; however, on a long time average, the resulting entropy increase will be positive, as the hotter subdomain must emit a higher energy (higher frequency) packet to the cooler one with greater probability, and vice versa. This is, by some means, similar to the thermodynamic limit.

### 4.2. Spin-Lattice Relaxation

The spin–lattice relaxation is a mechanism in which the parallel component of the nuclear magnetic moment relaxes from a higher energy non-equilibrium state to the thermodynamic equilibrium. In the initial condition, the magnetic moment is antiparallel to the constant magnetic field and its temperature is equal to the temperature of the surrounding thermal bath. During the relaxation process, the energy difference of the Zeeman levels has to be considered

(43) $$\Delta E=\varepsilon =\gamma \hbar B_{0},$$

where γ is the gyromagnetic ratio, and $B_{0}$ is the external magnetic field. To apply the results obtained above, it is necessary to express the relevant frequency expression, such as the Larmor frequency, which is

(44) $$\omega =\gamma B_{0}\;\;\;\mathrm{or}\;\;\;\nu =\frac{1}{2\pi }\gamma B_{0}.$$

The entropy current of a single spin relaxation process, using the expression in Equation (31), can be formulated as

(45) $$I_{S}=\frac{\pi^{2}k_{B}}{3}\nu =\frac{\pi k_{B}\gamma B_{0}}{6}.$$

The entropy production during the relaxation process can be obtained by the application of Equation (35), which results in

(46) $$\Sigma =\frac{1}{T}\frac{\pi^{2}}{3}h\nu^{2}=\frac{1}{T}\frac{1}{12}h\gamma^{2}B_{0}^{2}.$$

It is worth noting that the obtained quantity is quadratic in both the gyromagnetic ratio and the external magnetic field. This means that manipulation of nuclei with high γ results in higher entropy production. The temperature dependence of 1/T is somewhat expected.

These results may be useful to understand, in general, the spin relaxation, basic thermodynamic relations in spintronics to achieve the minimal loss of spin-waves [34], and in magnetic resonances [35], in the study of magnetic storage systems or quantum computing [36]. Similar considerations can be made for any process with relaxation or interaction with light, e.g., for photoluminescence.

## 5. An Additional Consequence of the Least Action Principle

Let us turn back to the action principle of heat conduction. We could see that the action (expressed by the temperature applying Equations (19) and (22))

(47) $$\tilde{S}\left(t\right)=\int_{0}^{t}\frac{1}{2}{\left(T-T_{0}\right)}^{2}\;dt$$

is the extremum (minimum) of the equalization process, i.e., it is minimal for the realizable motion. As it can be recognized from Equation (29), it pertains to the entropy transfer, thus the transferred entropy should be minimum for the realistic motion. Furthermore, this also means that the transferred energy during time *t* is also related to the minimal entropy conductance. The factor, $\Lambda_{s}$, appeared in Equation (29) and multiplied by the action in Equation (47) returns a quantity that has the unit of energy. Considering the aforementioned reasoning, the obtained quantity yields

(48) $$\tilde{E}=\Lambda_{s}\tilde{S}\left(t\right)=\frac{\pi^{2}k_{B}^{2}}{3h}\int_{0}^{t}\frac{1}{2}{\left(T-T_{0}\right)}^{2}\;dt,$$

which corresponds to the transferred energy during the process. Finally, we conclude that the above-formulated action principle is equivalent to the minimal entropy transfer of time-dependent (non-stationary) processes. The formulated relations bring us closer to both the understanding of entropy current conductance and, eventually, to the meaning of the Lagrangian formulation of the thermal process.

## 6. Summary

It is increasingly essential to understand the irreversibility of quantum mechanical and quantized transport processes on a microscopic scale. It is pointed out that both quantized entropy current and entropy production can be introduced and interpreted during the transfer of a single energy quantum. This completes the thermodynamic description of the process, including the validity of the second law of thermodynamics. Integrating into the theoretical framework of the least action principle on thermal propagation, it became apparent that this principle expresses the minimal entropy transfer on a quantum scale. Moreover, we pointed out that the description of the minimum entropy transfer principle is equivalent to the Lagrangian description of thermodynamics on the nanoscale. We believe that this new result is useful in the field of quantum computing, understating how information loss can occur and thus how it can be tackled.

## 7. Conclusions

Understanding the dissipation of the microscopic and nanoscale world is currently a major scientific challenge. The quantized entropy conductance introduced is a remarkably important point for clarifying the dissipation of heat transport on the quantum scale. Our result shows that the entropy change during a single phonon transfer is proportional to the square of the quasi-particle frequency. Based on these results, we aim to establish a relationship between quantum decoherence and thermodynamic background in the future. We believe our work could aid further studies understanding the microscopic world and might find applications in the field of quantum computing and/or spintronics.
